# Randomized trial of medroxyprogesterone acetate for the prevention of endometrial pathology from adjuvant tamoxifen for breast cancer: SWOG S9630

**DOI:** 10.1038/npjbcancer.2016.24

**Published:** 2016-08-10

**Authors:** Ronald K Potkul, Joseph M Unger, Robert B Livingston, Katherine D Crew, Sharon P Wilczynski, Caryl G Salomon, Barbara L Smith, Lucas Wong, David L Campbell, David E Einspahr, Garnet L Anderson, Dawn Hershman, Gary E Goodman, Powel H Brown, Frank L Meyskens, Kathy S Albain

**Affiliations:** 1 Loyola University Chicago Stritch School of Medicine, Cardinal Bernardin Cancer Center, Maywood, IL, USA; 2 SWOG Statistical Center, Seattle, WA, USA; 3 Arizona Cancer Center, Tucson, AZ, USA; 4 Columbia University, New York, NY, USA; 5 City of Hope, Duarte, CA, USA; 6 Massachusetts General Hospital Cancer Center, Boston, MA, USA; 7 Scott & White Memorial Hospital, Temple, TX, USA; 8 University of California at Davis Affiliate, Sierra Nevada Memorial Hospital, Grass Valley, CA, USA; 9 Stormont-Vail HealthCare, Topeka, KS, USA; 10 Swedish Cancer Institute/Pacific Cancer Research Consortium NCORP, Seattle, WA, USA; 11 MD Anderson Cancer Center, Houston, TX, USA; 12 University of California at Irvine, Chao Family Comprehensive Cancer Center, Orange, CA, USA

## Abstract

The proliferative effect of adjuvant tamoxifen on the endometrium can potentially result in endometrial abnormalities, including cancer in postmenopausal women. We conducted a randomized, controlled trial to assess endometrial pathological diagnoses in postmenopausal women with early stage, ER-positive breast cancer without endometrial pathology at baseline. They were assigned to tamoxifen alone versus tamoxifen plus cyclical medroxyprogesterone acetate (MPA 10 mg for 14 days every 3 months) for 5 years. Endovaginal sonograms (EVS) +/− endometrial biopsies (EMB) were required at baseline, 2 and 5 years. Of 313 patients registered, 296 were eligible and 169 (57%; 89, tamoxifen; 80, tamoxifen+MPA) were evaluable (completed year-2 EVS, with an EMB if stripe width was ⩾5 mm). Sixty (67%) of these in the tamoxifen arm had an endometrial stripe width ⩾5 mm (and underwent subsequent EMB) compared with 48 (60%) in the tamoxifen+MPA arm (*P*=0.40). There were four cases of proliferative endometrium and one simple hyperplasia on the tamoxifen arm (6% (95% confidence interval (CI): 2–13%) among evaluable patients and one proliferative endometrium on the tamoxifen+MPA arm (*P*=0.11). The overall fraction with benign endometrial abnormalities at year 2 was 3.6% (6/169; 95% CI: 1.3–7.6%), with only 1 (of 102) new benign proliferative event at year 5. The event rate in both arms was much lower than projected, making treatment arm comparisons less informative. A normal endometrium prior to tamoxifen may provide reassurance regarding future endometrial events. However, validation in a larger trial is needed before changing practice in asymptomatic, postmenopausal women.

## Introduction

The selective estrogen receptor modulator tamoxifen is one of the most frequently prescribed anticancer therapies worldwide. It is highly efficacious in the adjuvant setting for early stage, estrogen receptor-positive breast cancer as well as in the chemoprevention of breast cancer for patients at high risk.^1–3^ Yet, a significant number of patients with breast cancer as well as the healthy high-risk population choose to forego these benefits, often based on concern over the risk of endometrial cancer, a known but uncommon side effect of tamoxifen.

The initial report of Killackey *et al*.^4^ in 1985 suggested a possible link between tamoxifen use and the development of endometrial cancer. Since then, other studies reported tamoxifen’s association with endometrial pathologies—including hyperplasia, polyps, carcinoma, and sarcoma—in up to 36% of postmenopausal women taking tamoxifen for breast cancer.^5–8^ A large phase III trial, National Surgical Adjuvant Breast and Bowel Project (NSABP) B-14, found a 7.5-fold increase in the risk of endometrial cancer in the tamoxifen-treated arm versus the placebo group, with an average annual hazard ratio of 0.2/1000 for placebo versus 1.6/1000 in the tamoxifen arm.^9^ This risk of endometrial cancer was substantiated in the Oxford Overview, which also found that the risk increased as the duration of tamoxifen therapy increased.^10^

Unlike its anti-estrogenic effect in the breast, tamoxifen acts as a weak estrogen agonist on the endometrium, resulting in an increased risk of estrogen-like changes compared with non-users.^5,6,11,12^ These tamoxifen-associated changes include postmenopausal proliferative endometrium and adenomatous hyperplasia with or without atypia at rates as high as 30% compared with untreated patients.^13–15^ Endometrial hyperplasia was also found to be more common among healthy women who received preventive tamoxifen compared with untreated women.^16^ On the basis of these findings, a shift to recommend annual assessments of the uterine lining by endovaginal sonogram (EVS) and endometrial biopsy (EMB) during tamoxifen therapy was prevalent at the time this study was designed.

The induction of endometrial abnormalities by estrogen can be attenuated by the addition of progestin among women receiving hormone replacement therapy.^17^ We hypothesized that cyclical use of a progestin in women receiving tamoxifen might decrease the rate of both benign and malignant endometrial neoplasia. At the time of study design, medroxyprogesterone acetate (MPA) was the most commonly prescribed progestin in the United States. A quarterly schedule of 10 mg MPA for 14 days was equivalent to 5–10 mg progestin monthly in reducing the rate of endometrial hyperplasia in patients receiving estrogen replacement.^18^

The objectives of this randomized controlled trial for postmenopausal women about to embark on adjuvant tamoxifen was to compare the addition of cyclical MPA at the same time as tamoxifen compared with the control arm of tamoxifen alone, and to assess endometrial pathology rates via EVS/EMB in both arms at year 2 and year 5.

## Results

### Accrual and eligibility

A total of 313 patients were registered to the study from March 1997 to December 2004. Seventeen patients were ineligible due to: baseline endometrial stripe width ⩾5mm with proliferative changes on EMB (7); active breast cancer at registration (5); T4 disease (3); and tamoxifen started >28 days prior to registration (2). Median follow-up among patients still alive was 9.0 years (maximum 12.9 years). Among the 296 eligible patients, 149 received tamoxifen alone and 147 received tamoxifen+MPA.

### Patient characteristics

Patient characteristics are shown on Table 1. The median age was 59.5 years. Most patients were white (92%), with 3% African American. 3% Asian and 2% other. Thirty-six percent of patients received prior adjuvant chemotherapy. About one quarter of eligible patients had baseline stripe width ⩾5 mm, on the pre-study EVS, among whom the predominant EMB findings were benign polyp or other benign findings (84%) or no tissue on EMB. There was no significant difference between arms in any patient characteristic or in percent of patients evaluable for the year-2 primary end point.

### Treatment delivered and toxicity

Mean time on treatment was 3.7 (s.d.=1.7) years for patients on the tamoxifen arm and 3.6 (s.d.=1.9) years on the tamoxifen+MPA arm (*P*=0.79). Time to event analysis showed that the probability of being on treatment at 2 years was 81% for patients on the tamoxifen arm and 73% for patients on the tamoxifen+MPA arm (hazard ratio: 1.47; 95% confidence interval (CI): 0.90–2.39, *P*=0.12).

All protocol-specified treatment was completed as planned for 53% of patients overall (50% on the tamoxifen arm and 55% on the tamoxifen+MPA arm, *P*=0.41). Removal from protocol treatment owing to toxicity or worsening illness occurred for 16 patients (11%) on the tamoxifen alone arm and 20 patients (14%) on the tamoxifen+MPA arm (*P*=0.56; Table 2). Two patients on the tamoxifen arm and 11 patients on the tamoxifen+MPA arm received incorrect or no treatment. These patients were not evaluated for toxicity.

On the tamoxifen arm, 147 patients were evaluated for toxicity. One patient died from a myocardial infarction judged to be treatment-related. Two patients had grade 4 depression. Twenty-one patients had at worst grade 3 toxicity. On the tamoxifen+MPA arm, 136 patients were evaluated for toxicity. One patient died from a pulmonary embolism judged to be treatment-related. One patient had grade 4 hypercholesterolemia. Twenty-six patients had at worst grade 3 toxicity. There was no significant difference in the proportion of patients with greater that or equal to grade 3 toxicity by arm (*P*=0.44).

### Primary end point: endometrial pathological diagnosis at year 2

**Figure 1:**
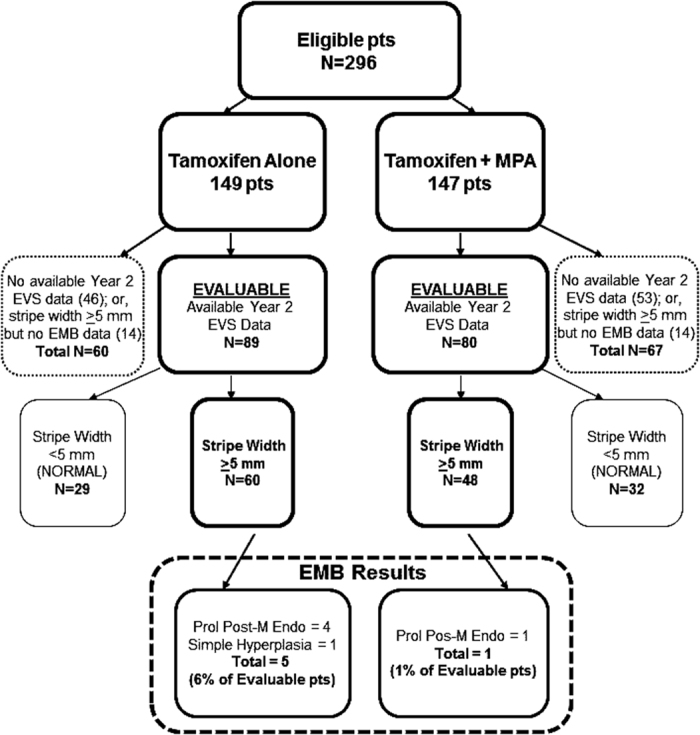
Year-2 results. EMB, endometrial biopsies; EVS, endovaginal sonograms; MPA, medroxyprogesterone acetate; pts, patients.

Of 313 patients registered, 296 were eligible and 169 (57%) were fully evaluable (defined as completing the year-2 EVS and undergoing EMB if stripe width was ⩾5 mm). By arm, 89 (60%) on tamoxifen and 80 (54%) on tamoxifen+MPA were fully evaluable (Figure 1). Of these evaluable patients, 60 (67%) in the tamoxifen arm had an endometrial stripe width ⩾5 mm (and underwent subsequent EMB) compared with 48 (60%) in the tamoxifen+MPA arm (*P*=0.40). On the tamoxifen arm, 84 of 89 evaluable patients (94%) were scored as “normal” due to stripe width <5 mm (*n*=29) or benign EMB results such as polyps (*n*=55). The other 5 evaluable patients (6%; 95% CI: 2–13%) had abnormalities including proliferative postmenopausal endometrium (*n*=4) and simple hyperplasia (*n*=1). On the tamoxifen+MPA arm, 79 of the 80 fully evaluable (99%) were scored “normal” due to stripe width <5 mm (*n*=32) or benign EMB results (*n*=47). One evaluable patient (1%; 95% CI: 0.1–7%) had proliferative postmenopausal endometrium. There were no differences by arm in the proportion of patients with stripe width <5 mm (33% vs. 40%, respectively; *P*=0.40). Overall, 6 of the 169 evaluable patients (3.6%; 95% CI: 1.3–7.6%) had abnormal endometrial biopsies, none of which were high-risk lesions. In a logistic regression model adjusting for baseline stratification factors, there was no difference by arm in endometrial pathological diagnosis at year 2 (OR=0.15; 95% CI: 0.01–1.54, *P*=0.11).

A sensitivity analysis considered the possible impact of the criteria used for determination of evaluability on the pathological diagnosis rate in the tamoxifen arm. Sixty out of 149 eligible patients were unevaluable owing to no available year-2 EVS data or stripe width ⩾5mm, but no EMB data submitted. If, conservatively, the rate of pathological diagnosis in unevaluable patients was 30%, the anticipated rate by design, then an additional 18 cases with pathological diagnosis would have been identified (0.3×60) and the overall rate among all eligible patients would be 15% (23/149) with an upper confidence limit of 22%. The rate of pathological diagnosis among the unevaluable cases would have had to be 48% (29/60) before the upper confidence limit for the overall rate of pathological diagnosis overlapped with the predicted rate of 30%. Therefore, the exclusion of unevaluable patients is highly unlikely to explain the low observed rate of pathological diagnosis in the tamoxifen arm.

### Pathological diagnosis at year 5 (a secondary end point)

**Figure 2:**
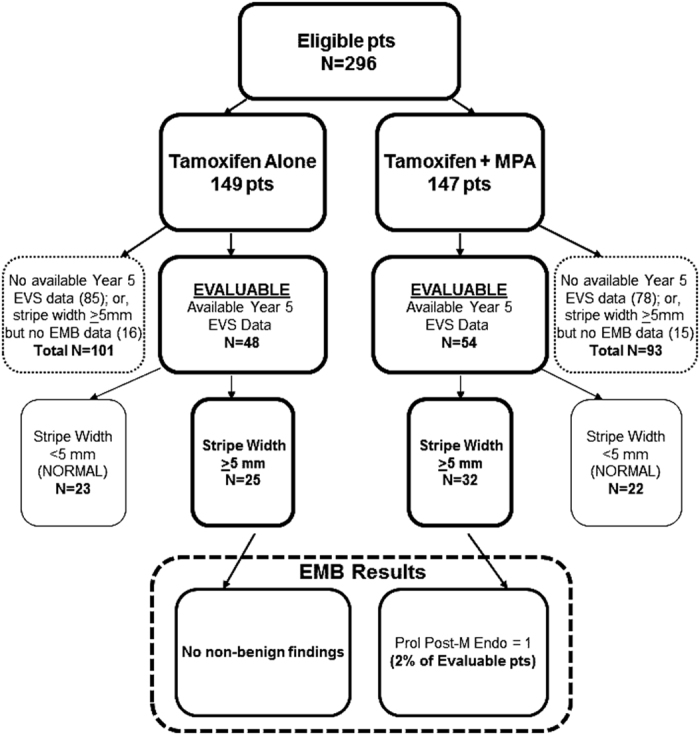
Year-5 results. EMB, endometrial biopsies; EVS, endovaginal sonograms; MPA, medroxyprogesterone acetate; pts, patients.

Year-5 data (Figure 2) were available for the evaluable subset (EVS data available) of 102 patients, 48 on the tamoxifen arm and 54 on the tamoxifen+MPA arm. The endometrial stripe width was ⩾5 mm in 25 (52%) on the tamoxifen arm and 32 (59%) on the tamoxifen+ MPA arm (*P*=0.47, Figure 2). Only a single finding was identified: one patient on the tamoxifen+MPA arm had a proliferative endometrium.

### Analysis of adverse outcomes

Summary data on number of adverse outcomes are shown in Table 2. In total, 40 patients (27%) on the tamoxifen arm had at least one of the specified adverse outcomes, compared with 35 (24%) on the tamoxifen+MPA arm (*P*=0.55). When adverse events were limited to breast cancer progression/death or positive EMB results, representing explicit manifestations of malignancy, the rates were 18% and 12%, respectively (*P*=0.11).

## Discussion

The addition of quarterly MPA to tamoxifen was feasible with minimal toxicity in this prospective, randomized trial of postmenopausal women with an intact uterus eligible to receive standard adjuvant tamoxifen for 5 years. There was no statistically significant difference in endometrial pathology after 2 years (5 vs. 1 for tamoxifen and tamoxifen+MPA, respectively *P*=0.11). However, a striking observation was the low event rate in the tamoxifen arm; specifically, the fraction of patients with abnormal endometrial pathology (6%), including its upper 95% confidence limit (13%), was much lower than the projected 30% in the trial design.^13–15^ Concern about endometrial cancer risk is a major reason that women with early stage, endocrine responsive breast cancer, non-invasive disease, or increased breast cancer risk decline their physician’s recommendation for tamoxifen.^19–21^

Furthermore, all five potentially estrogen-related abnormal pathological findings at year 2 in the tamoxifen arm were low-grade conditions (four proliferative endometrium and one simple hyperplasia without atypia). There was no evidence of premalignant endometrial findings or carcinoma at any time point in this study. This low incidence of pathological diagnosis overall is most likely attributable to the eligibility requirement of a pre-study EVS with a normal stripe width or normal endometrial pathology if a thickened stripe was identified before enrollment. Although annual “screening” of women on tamoxifen with EVS and EMB is no longer recommended, these data support offering a baseline EVS (followed by an EMB if the stripe width is increased) to postmenopausal women concerned about the risk of endometrial pathology. A baseline normal endometrium prior to tamoxifen may provide additional reassurance about endometrial safety with tamoxifen therapy.

Many risk factors such as nulliparity, early onset of menarche, late onset of menopause, obesity, and diabetes are shared by patients with either endometrial or breast carcinoma. Thus, it is not surprising that breast cancer patients also have a higher risk of endometrial cancer and its associated precursors. In one study, menopausal women with estrogen receptor-positive breast cancer appeared to have a high risk of baseline subclinical endometrial abnormalities.^22^ Thirty-seven percent of patients had a thickened endometrial stripe and 11%, 4%, and 3% were found to have endometrial polyps, simple hyperplasia, and complex atypical hyperplasia, respectively. In such patients, increased endometrial stimulation by tamoxifen might be superimposed on occult endometrial disease leading to increased risk of subsequent endometrial cancer while on tamoxifen. This group of patients was eliminated in this trial by requiring a normal EVS or if abnormal, a normal EMB.

Two other studies provide supporting evidence for the importance of ruling out pre-existing endometrial pathology in postmenopausal women about to embark on adjuvant tamoxifen therapy.^23,24^ In one study, high-risk women were identified in whom endometrial abnormalities were diagnosed before commencement of tamoxifen therapy.^23^ Despite reversing these lesions prior to instituting tamoxifen, these patients still had a significantly higher rate of subsequent endometrial abnormalities following 3 years of tamoxifen compared with similar patients who had no endometrial abnormalities before initiating tamoxifen therapy. The incidence of subsequent atypical lesions was significantly higher in women with endometrial abnormalities initially than those without (3/9 versus 1/51, *P*=0.009). This rate in the group without lesions at the beginning of tamoxifen therapy is similar to what we encountered in our control group. The other study demonstrated that obesity and estrogen replacement therapy significantly modify the association between tamoxifen use and endometrial cancer risk.^24^ The authors recommended that these patients merit closer surveillance during tamoxifen than those without these risk factors.

Our study has several important limitations. The low rate of endometrial pathology in the study overall prevents an adequate assessment of the value of prophylactic cyclical MPA during tamoxifen therapy. The study was not blinded and a large number of patients were not fully evaluable at year 2 owing to the missing data. The number of patients who did not complete planned therapy in each arm added to the smaller numbers at year 2 and especially year 5, although poor adherence and compliance to endocrine therapy is a universal problem with both tamoxifen and aromatase inhibitor adjuvant therapy.^19^ Although aromatase inhibitors are now the standard of care for the treatment of postmenopausal hormone-sensitive breast cancer, chronic side effects such as arthralgia and osteoporosis can impact compliance and are more common than endometrial pathology with tamoxifen.^25–28^ Furthermore, our study was not powered to assess the effect of adding MPA to tamoxifen on other chronic disease end points known to be affected by either agent.^3,29^

In summary, our results suggest that evaluation of the endometrium might be considered for certain postmenopausal women about to receive tamoxifen in the adjuvant or chemoprevention setting if there are concerns about developing endometrial abnormalities during tamoxifen. In the group with a negative pre-tamoxifen uterine evaluation, the risk of developing malignancies or precursors to malignancies appears to be extremely low, but these findings should be viewed as hypothesis generating due to the small sample size of this randomized, controlled trial. Nevertheless, overall we observed only 6 abnormal endometrial biopsy results among 169 evaluable patients (3.6%; 95% CI: 1.3–7.6%) at year 2, with just 1 (of 102) additional benign proliferative event at year 5. None of these abnormalities were high-risk lesions. These data could potentially reassure certain postmenopausal women who fear endometrial side effects of tamoxifen, but would otherwise benefit from instituting this important adjuvant breast cancer and breast cancer chemoprevention therapy. However, validation in a larger trial is needed first before changing practice in all asymptomatic postmenopausal women.

## Materials and methods

Postmenopausal women with newly diagnosed primary invasive estrogen receptor-positive adenocarcinoma of the breast, ductal carcinoma *in situ*, lobular carcinoma *in situ* with microinvasion, or Paget’s disease of the nipple were eligible (stages Tis-3, N0-1, M0). Standardized National Cancer Institute intergroup criteria were used to confirm postmenopausal status. Prior tamoxifen up to 28 days was permitted and any adjuvant chemotherapy if given had to be completed. Post-lumpectomy radiation therapy was required.

A pelvic examination with a pap smear less than a year prior to registration was required, with a baseline EVS within 3 months prior to registration. If the endometrial stripe was ⩾5 mm on the EVS, an EMB was required. Patients with either inadequate tissue (considered a normal finding in postmenopausal women) or benign pathology such as a polyp were eligible. Any baseline biopsy with proliferative changes, hyperplasia, or carcinoma rendered the patient ineligible. The protocol was approved by the Institutional Review Board at each participating site. Written consent was obtained from participants before enrollment. The study was conducted in accordance with the Declaration of Helsinki and was registered with ClinicalTrials.gov (NCT00002920).

Patients were randomized to adjuvant therapy with tamoxifen alone or tamoxifen plus medroxyprogesterone acetate (MPA). Randomization assignment was stratified according to (1) adjuvant chemotherapy, yes vs. no; and (2) number of positive nodes, 0–3 vs. ⩾4. Tamoxifen was given at 20 mg orally per day for 5 years, with added MPA 10 mg orally per day for 14 days every 3 months for 5 years in the experimental arm. The study was not blinded.

Toxicity was assessed every 3 months during treatment. An EVS was required at the conclusion of year 2 (±3 months) and at the end of year 5 of tamoxifen or tamoxifen+MPA treatment. If the endometrial stripe on the EVS was ⩾5 mm, an EMB was mandated. Endometrial biopsies were performed within ±6 months of the 2-year end point and within ±1 year of the 5-year end point.

The primary clinical end point was the rate of abnormal endometrial biopsy findings 2 years after randomization, with rates compared between the arms. “Normal” was defined as an endometrial stripe <5 mm on EVS or a biopsy revealing no tissue or other benign findings such as polyps. “Abnormal” was an endometrial pathological diagnosis of either proliferative change, simple or cystic hyperplasia, complex (adenomatous) hyperplasia, hyperplasia with atypia, or carcinoma. Secondary end points included the same endometrial pathology definitions at the conclusion of 5 years of tamoxifen or tamoxifen+MPA, the comparison of endometrial thickness and uterine pathology, assessment of treatment toxicity (i.e., adverse events), and the number of adverse outcomes at any time between registration and the conclusion of the 5-year time window, defined as: (1) evidence of positive diagnosis according to endometrial biopsy at 2 years or 5 years; (2) evidence of dropout related to worsening health or toxicity; and (3) evidence of breast cancer progression or death.

The original study design anticipated 208 eligible patients would be required to achieve 176 patients evaluable (88 per arm) for the 2-year primary end point (i.e., 15% dropout at 2 years). This required about 230 patients registered under a typical 10% ineligibility rate. The study was subsequently revised to raise the accrual goal given a higher than anticipated observed 2-year dropout rate. The revised accrual goal required 330 patients in order to achieve the evaluable accrual goal of 176. Evaluable patients must have met the eligibility criteria and had a 2-year EVS with either an endometrial stripe <5 mm, or, if the stripe was ⩾5 mm, had an endometrial biopsy.

The design projected a 30% rate of endometrial pathological diagnoses on the observation arm, based on the assumption that 40% of patients will have attempted biopsies at 2 years that yield material, and 75% of these cases will have an endometrial pathological diagnosis.^16^ Therefore, 176 patients would provide sufficient power to detect a 75% reduction in endometrial pathological diagnosis, from 30% in the observation arm to 7.5% on the MPA arm, with 96% power, given an *α*=0.05 two-sided test. The primary analysis was based on logistic regression, adjusting for the baseline stratification factors.

## Figures and Tables

**Table 1:** Characteristics of eligible randomized patients and year-2 evaluable patients by treatment arm

*Characteristics*	*Eligible randomized*			*Evaluable for year-2 end point*		
	*Tamoxifen* N*=149*	*Tamoxifen+MPA* N*=147*	P *value*	*Tamoxifen* N*=89*	*Tamoxifen+ MPA N=80*	P* value*
Median age (years)	59.2	59.8	0.63	58.8	59.0	0.91
Prior adjuvant chemotherapy	54 (36%)	53 (36%)	0.97	34 (38%)	32 (40%)	0.81
⩾4+ nodes	5 (3%)	10 (7%)	0.18	2 (2%)	7 (9%)	0.06
*Race*						
White	138 (93%)	135 (92%)	0.80	87 (98%)	78 (98%)	0.91
African American	4 (3%)	4 (3%)		0	0	
Asian	4 (3%)	5 (3%)		1 (1%)	2 (3%)	
Other/unknown	3 (2%)	3 (2%)		1 (1%)	0	
Obese, BMI⩾30 kg/m^2^	46 (31%)	50 (34%)	0.56	23 (26%)	27 (34%)	0.26

Abbreviations: BMI, body mass index; MPA, medroxyprogesterone acetate.

**Table 2:** Adverse outcomes by treatment arm in eligible randomized patients

*Outcome variable*	*Arm 1*	*Arm 2*
	*Tamoxifen alone* N*=149*	*Tamoxifen+MPA* N*=147*
Breast cancer progression or death	23 (15%)	14 (10%)
Removal from protocol owing to toxicity or worsening illness	16 (11%)	20 (14%)
Positive EMB	5 (3%)	3 (2%)
Total (any event)	40 (27%)	35 (24%)
Total (Positive EMB, progression, or death)	27 (18%)	17 (12%)

Abbreviations: EMB, endometrial biopsy; MPA, medroxyprogesterone acetate.

Patients may have experienced more than one adverse outcome.
